# Novel Graphene Biosensor Based on the Functionalization of Multifunctional Nano-bovine Serum Albumin for the Highly Sensitive Detection of Cancer Biomarkers

**DOI:** 10.1007/s40820-019-0250-8

**Published:** 2019-03-09

**Authors:** Lin Zhou, Kun Wang, Hao Sun, Simin Zhao, Xianfeng Chen, Dahong Qian, Hongju Mao, Jianlong Zhao

**Affiliations:** 1 State Key Laboratory of Transducer Technology; Key Laboratory of Terahertz Solid-State Technology, Shanghai Institute of Microsystem and Information Technology, Chinese Academy of Sciences, Shanghai, 200050 People’s Republic of China; 2 School of Biomedical Engineering, Shanghai Jiao Tong University, Shanghai, 200240 People’s Republic of China; 3 School of Electronic Engineering, Bangor University, Bangor, LL57 1UT UK

**Keywords:** Bio-interface, Multifunctional denatured BSA, GFET biosensor, Cancer biomarker

## Abstract

A simple, convenient, and highly sensitive bio-interface for graphene field-effect transistors (GFETs) based on multifunctional nano-denatured bovine serum albumin (nano-dBSA) functionalization was developed to target cancer biomarkers. The novel graphene–protein bioelectronic interface was constructed by heating to denature native BSA on the graphene substrate surface. The formed nano-dBSA film served as the cross-linker to immobilize monoclonal antibody against carcinoembryonic antigen (anti-CEA mAb) on the graphene channel activated by EDC and Sulfo-NHS. The nano-dBSA film worked as a self-protecting layer of graphene to prevent surface contamination by lithographic processing. The improved GFET biosensor exhibited good specificity and high sensitivity toward the target at an ultralow concentration of 337.58 fg mL^−1^. The electrical detection of the binding of CEA followed the Hill model for ligand–receptor interaction, indicating the negative binding cooperativity between CEA and anti-CEA mAb with a dissociation constant of 6.82 × 10^−10^ M. The multifunctional nano-dBSA functionalization can confer a new function to graphene-like 2D nanomaterials and provide a promising bio-functionalization method for clinical application in biosensing, nanomedicine, and drug delivery.

**Electronic supplementary material:**

The online version of this article (10.1007/s40820-019-0250-8) contains supplementary material, which is available to authorized users.

## Highlights

- A simple and convenient graphene bio-interface was designed by using multifunctional nano-denatured bovine serum albumin (nano-dBSA) film.
- Highly sensitive cancer biomarker detection in diluted serum at the femtogram per milliliter level was achieved using the nano-dBSA functionalized graphene field-effect transistor.

**Electronic supplementary material:**

The online version of this article (10.1007/s40820-019-0250-8) contains supplementary material, which is available to authorized users.

## Graphical abstract

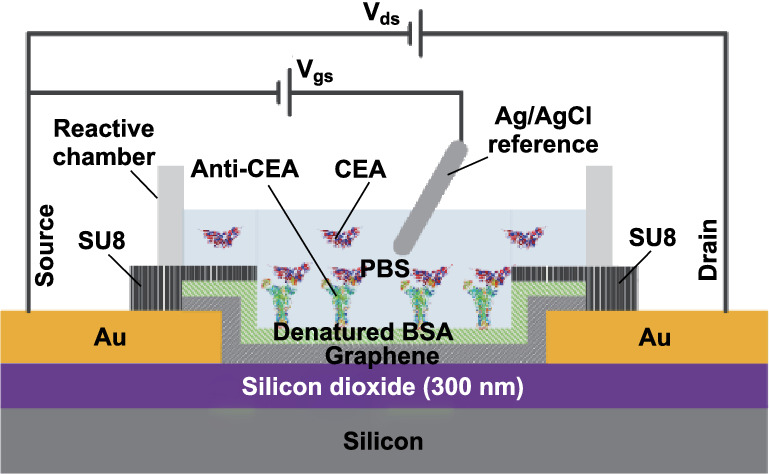

## Introduction

Cancer is a major public health problem worldwide. For many cancers, it can take 20–30 years for initial lesions to progress to late-stage disease [1]. Early detection is the key to cancer control, especially in reducing incidence rates and cancer-related deaths [2]. Cancer protein biomarkers have been widely used in the early diagnosis of cancer. Carcinoembryonic antigen (CEA) is one of the most commonly used specific blood-based biomarkers for clinical tumor diagnosis. CEA is routinely used as an important indicator in annual medical checkups in many countries [3]. Serum CEA concentration is closely correlated with malignant tumors, such as colorectal cancer [4], gastric cancer [5], medullary thyroid cancer [6], lung cancer [7], and pancreatic carcinoma [8]. Determination of CEA concentration in a clinical sample can provide information about the severity of disease, tumor stage, pathological type, tumor metastasis, prognosis, and recurrence. Thus, CEA detection is valuable for the early diagnosis of cancer and has spurred efforts to develop strategies for the highly sensitive detection of CEA. The different strategies include photoelectrochemical immunosensors [9], time-resolved fluoroimmunoassay [10], surface-enhanced Raman scattering [11], fluorescence resonance energy transfer biosensors [12], electrochemical immunosensors [13], and electrochemiluminescence immunosensors [14, 15]. However, the development of a simple, low-cost, label-free, and rapid monitoring platform for the detection of cancer biomarkers for clinical diagnosis and screening applications remains a compelling goal.

Electrical detection of biomolecules based on their intrinsic charges is an efficient and ultrasensitive detection approach. Specifically, field-effect transistor (FET) biosensors are attractive because of their portability, inexpensive mass production, low power consumption, label-free detection, rapid response, and the potential for on-chip integration of the sensor and the electronic measurement system [16, 17]. In a FET biosensor, specific receptors immobilized in the sensing channel selectively capture the desired target biomolecules. The captured charged biomolecules can generate a doping or gating effect on the channel [18–21]. Both are converted into a readable electrical signal by the FET, usually as a drain-to-source current or channel transconductance.

Interfacing biomolecules with channel sensing materials is a critical challenge to fabricate high-performance and inexpensive FET biosensors [22, 23]. In particular, the emergence of two-dimensional (2D) nanomaterials, such as graphene [24–26], molybdenum disulfide [27, 28], and black phosphorus [29], offers new powerful diagnostic tools for in vitro diagnosis and biomedical science applications. Graphene and graphene derivatives have been widely used in protein biomarker detection because of their tunable optical properties, high specific surface area, good biocompatibility, and easy functionalization [30–34]. Furthermore, the ambipolar field-effect, exceptional electrical properties, and atomically thin structures make graphene very promising as a channel material for FET biosensors [35], because of its excellent electrostatic coupling with charged target biomolecules.

The specificity and action of these biosensors depend on the coupling of effective recognition components on the graphene surface through noncovalent interactions that will not damage the graphene lattice or degrade its electronic performance. Noncovalent linkers mainly exploit π-stacking interactions and hydrophobic forces to attach directly on the graphene surface [36]. Bifunctional noncovalent linkers, such as 1-pyrenebutanoic acid succinimidyl ester, *N*-hydroxysuccinimide (NHS) ester tripod, bovine serum albumin (BSA), pyrene butyric acid, and gold nanoparticles, have been successfully used to construct a bio-interface of graphene FET biosensors for the detection of glucose [37], DNA molecules [16], single-nucleotide polymorphisms [38], proteins [17, 39–41], and other biochemicals [42, 43]. Studies have focused on many difficult problems and topics. However, the complex and uncontrollable bio-interface of graphene FET channels remains a hurdle.

Herein, a simple and one-step method using multifunctional nano-denatured BSA (nano-dBSA) film to construct a graphene FET biosensor is described. The system enables the highly sensitive detection of cancer biomarkers. To construct the biosensor, native BSA protein solution was denatured by heating on graphene to form a layer that protected from unexpected destruction and surface contamination. At the same time, this nano-dBSA film could also serve as a cross-linker for the immobilization of anti-CEA monoclonal antibody (mAb). With the integration of the denaturation process into the fabrication of a graphene FET and the enriched chemical groups on the dBSA surface, one-step modification using 1-ethyl-3-(3-dimethylaminopropyl)-carbodiimide (EDC)/sulfo-NHS immobilized receptors on the graphene channel. In addition, enhanced sensitivity of the graphene FET biosensor was achieved by exploiting the dBSA modification method. Field-induced sensitivities to various CEA concentrations were observed, ultimately resulting in good specific recognition of CEA in diluted serum at an ultralow concentration of 337.58 fg mL^−1^. The cooperativity and strong affinity between CEA and anti-CEA mAb were estimated by the Hill model. The electric detection of the binding of CEA was interpreted to follow the Hill model for ligand–receptor interaction, indicating the negative cooperativity in binding between CEA and anti-CEA mAb with a dissociation constant of 6.82 × 10^−10^ M.

The demonstration of multifunctional nano-BSA chemical functionalization provides new functions for graphene-like 2D nanomaterials for further applications, such as biosensing, nanomedicine, imaging, cancer therapy, and drug delivery.

## Experimental

### Materials

Graphene films grown by chemical vapor deposition on copper foil were purchased from 2D Carbon (Changzhou, China). BSA was obtained from Sangon Biotech (Shanghai, China, Purity: > 96%). The EDC and sulfo-NHS cross-linkers were purchased from Sigma-Aldrich (Darmstadt, Germany). Anti-CEA mAb_1_ and anti-CEA mAb_2_ were purchased from Medix Biochemica (Kauniainen, Finland). CEA protein and squamous cell carcinoma (SCC) were obtained from Fitzgerald (Acton, MA, USA) and Linc-Bio Science (Shanghai, China), respectively. Cytokeratin-19-fragment (CYFRA21-1) was purchased from Calbioreagents (Foster City, CA, USA). Quantum dots (QDs) with an emission wavelength of 625 nm were from Jiayuan Quantum Dot Co. (Wuhan, China). 1-Pyrenebutyric acid *N*-hydroxysuccinimide ester (PYR-NHS) was obtained from AnaSpec (Fremont, CA, USA). Polydimethylsiloxane (PDMS) was used to fabricate the reactive chamber. Deionized water obtained from a Millipore-Q purification system (Millipore, Billerica, MA, USA) was used for the preparation of all solutions.

### Graphene Device Fabrication

Photoresist was used to define the drain/source electrode on a 300-nm SiO_2_/Si substrate, followed by the deposition of titanium and gold metals by electron beam evaporation. The metals on the photoresist were removed using acetone. The graphene-coated copper foil was etched using aqueous ammonium persulfate (10 g mL^−1^). Graphene was coated on the metal electrodes. Native BSA films were denatured on the graphene at 80 °C for 3 min, and the remaining dBSA/graphene films were etched using O_2_ plasma for 5 min. Finally, SU-8 photoresist was coated on the films as the insulating layer to prevent leakage current. The thickness of the dBSA functionalized graphene channel was optically characterized.

### Bioprobe Functionalization and Characterization

The functionalization of dBSA on the graphene was carried out in a reactive chamber. The concentration of anti-CEA mAb used for immobilization onto the dBSA film was 2 mg mL^−1^. The dBSA film was incubated with 5 mg mL^−1^ EDC, 1 mg mL^−1^ NHS, and anti-CEA mAb solution in the dark. After incubation, the remaining unconjugated antibodies were removed by rinsing with phosphate buffered saline (PBS). A 1% BSA solution was used to block the excess reactive groups remaining on the graphene surface for 1 h. Secondary anti-CEA mAb was labeled with QDs (100 nM) mixed with 100 ng mL^−1^ CEA solution. The mixed solution was incubated with anti-CEA-dBSA functionalized graphene and bare dBSA functionalized graphene for 1 h each. Finally, the fluorescent images of each dBSA functionalized graphene were recorded using a fluorescence microscope.

### Measurements

All electrical measurements were performed using a semiconductor parameter analyzer (Keithley 4200).

## Results and Discussion

### Fabrication of Nano-BSA Graphene FET

**Fig. 1:**
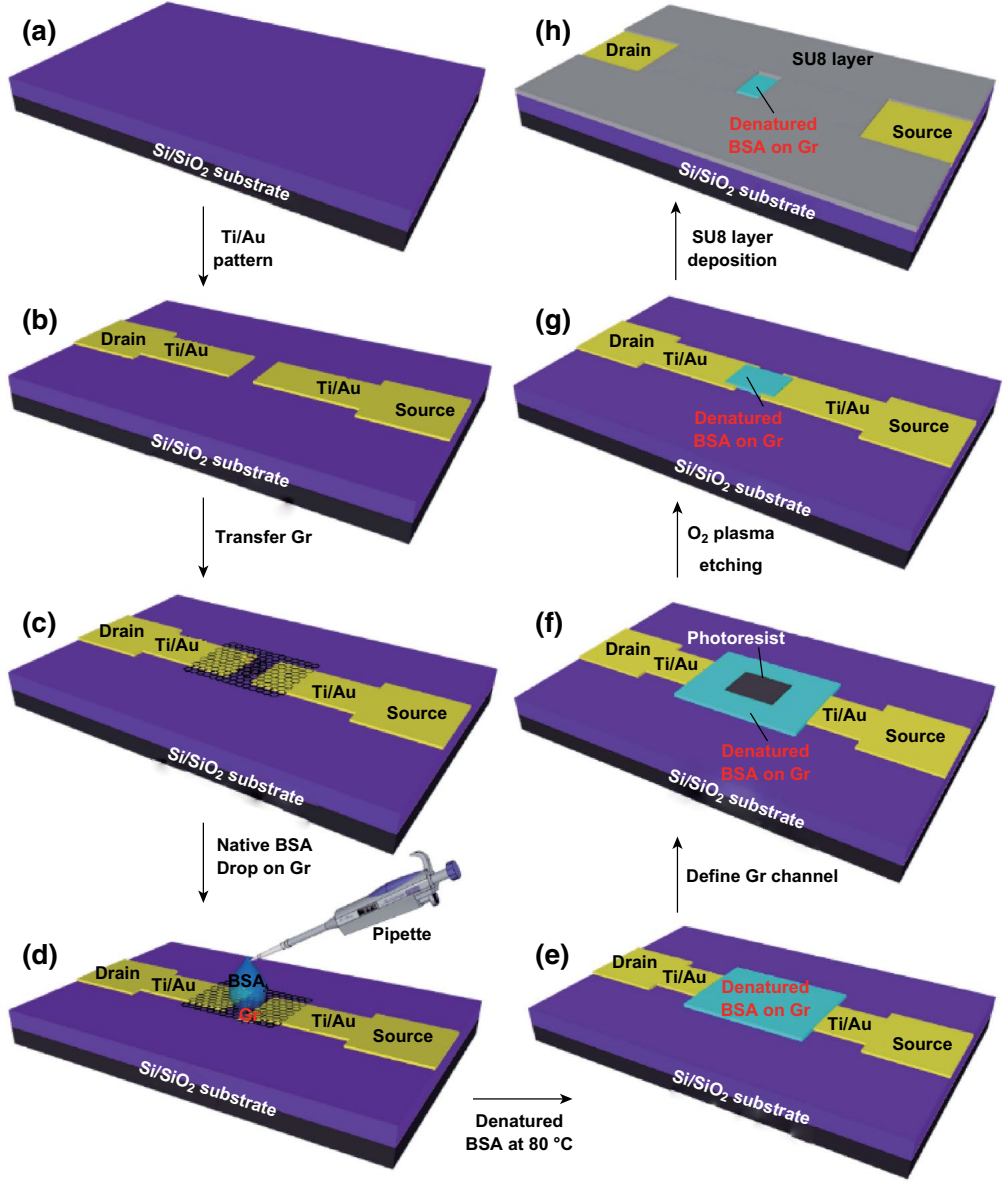
Fabrication of nano-dBSA functionalized GFET. **a** Silicon (Si)O_2_/Si substrate and **b** titanium/gold electrodes were patterned as source/drain electrode. **c** Graphene was transferred onto the metal electrodes. **d** Native BSA solution was dropped onto the graphene. **e** Native BSA was denatured on the graphene at 80 °C. **f** Photoresist was used to define the dBSA functionalized graphene channel. **g** O_2_ plasma was used to etch the dBSA film and graphene at the same time. **h** Substrate was coated with SU-8 photoresist as an insulating layer

Titanium and gold were patterned on the SiO_2_/Si substrate as source and drain electrodes by photolithography, metal deposition, and a liftoff process, as shown in Fig. 1a, b. Graphene grown by chemical vapor deposition was transferred to the metal electrodes on the substrate, and the poly(methyl methacrylate) film on the graphene was removed by acetone (Fig. 1c). Based on denatured BSA-doped graphene [39], native BSA dissolved in deionized water at a concentration of 1.5 mM was then dropped on the graphene (Fig. 1d) and the native BSA was denatured on the graphene surface at 80 °C, as shown in Fig. 1e. Obvious thin dBSA films were formed on graphene via noncovalent interactions, consistent with previous observations [44]. The dBSA functionalized graphene was defined by photolithography (Fig. 1f) and simultaneously etched using O_2_ plasma. The photoresist was removed by acetone (Fig. 1g). The dBSA functionalized graphene channel was 60 µm in width and 30 µm in length. The thickness of this channel measured optically was approximately 26.4 nm (Fig. S1). This multifunctional nano-dBSA film acted to prevent surface contamination and destruction of the graphene, and also functioned as a cross-linker between the graphene FET biosensor and bioconjugate receptor. Finally, to protect the gold contacts from the electrolyte and obviate the leakage current from these metal contacts to the electrolyte, a layer of chemically stable SU-8 photoresist was coated on the gold electrodes (Fig. 1h).

### Functionalization and Characterization of Bioprobes Based on Nano-dBSA Film

**Fig. 2:**
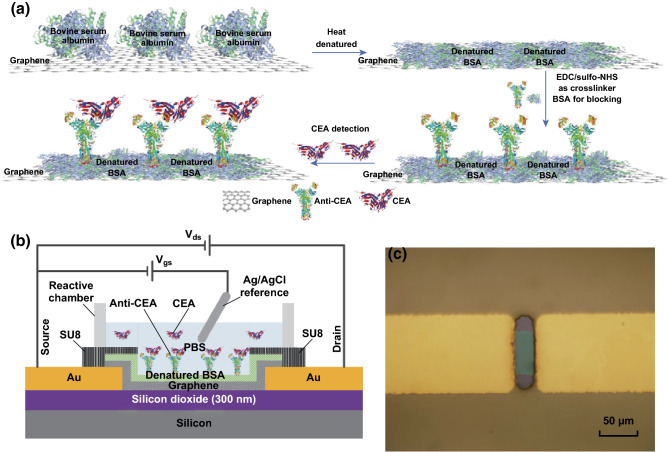
**a** Processes of the modification for anti-CEA-dBSA functionalized graphene. **b** Schematic diagram of electrolyte-gated anti-CEA-dBSA functionalized GFET. **c** Optical micrograph of the graphene channel

To achieve sensitive CEA recognition, the functionalized dBSA film enriched with chemical groups was used as a cross-linker of graphene. The film interacted with the graphene by π–π stacking. Anti-CEA mAb antibodies were conjugated onto the dBSA films via an immobilization procedure involving EDC and sulfo-NHS. EDC reacted with anti-CEA mAb to create an *o*-acylisourea intermediate, and a sulfo-NHS ester intermediate was formed by adding the sulfo-NHS, which could couple with amine-containing dBSA film on graphene. The resulting anti-CEA-dBSA functionalized graphene FETs (GFETs) acted as sensitive bio-interfaces to specifically recognize CEA. After immobilizing anti-CEA mAb and rinsing with PBS, native BSA solution was added to the channel of the dBSA functionalized GFET to block the excess reactive groups remaining on the dBSA surface. Finally, anti-CEA-dBSA functionalized GFETs were rinsed with deionized water and prepared for subsequent detection of target molecules. The entire process of the modification for anti-CEA-dBSA functionalized GFETs is shown in Fig. 2a.

Sandwich fluorescent immunoassay is a commonly used approach in biotechnology [45]. It was used to characterize the immobilization of anti-CEA mAb on dBSA film in this study. Secondary anti-CEA mAb conjugated with QDs was mixed with CEA solution and incubated with anti-CEA-dBSA functionalized graphene and bare dBSA functionalized graphene. Compared with the control group, the fluorescent images shown in Fig. S2 revealed that anti-CEA mAb was successfully immobilized on the dBSA functionalized graphene surface by the activation of EDC and sulfo-NHS. The results indicated that this novel method based on the dBSA film could be effective in the design of graphene biosensors.

### Construction of Electrolyte-Gated Anti-CEA-dBSA Functionalized GFET

The reaction chamber made of polydimethylsiloxane was anchored on the substrate using silicone. The miniaturized Ag/AgCl electrochemical reference electrode was immersed in the reactive chamber as the gate of the anti-CEA-dBSA functionalized GFET. Drain–source voltage (*V*_ds_) and gate–source voltage (*V*_gs_) were applied to force the operation of the devices. One terminal of the miniaturized Ag/AgCl electrochemical reference electrode was fixed on the shelf, and another terminal was immersed in the reactive chamber as the gate. Considering the sensitivity of anti-CEA-dBSA functionalized GFETs, 0.1 mM PBS was added to the reactive chamber as the electrolyte to maintain an appropriate Debye length [46]. A schematic diagram of electrolyte-gated anti-CEA-dBSA functionalized GFET is shown in Fig. 2b. In addition, a representative optical micrograph of the dBSA functionalized graphene channel with an SU-8 insulating layer is shown in Fig. 2c.

### Enhanced Performance of Anti-CEA-dBSA Functionalized GFET

**Fig. 3:**
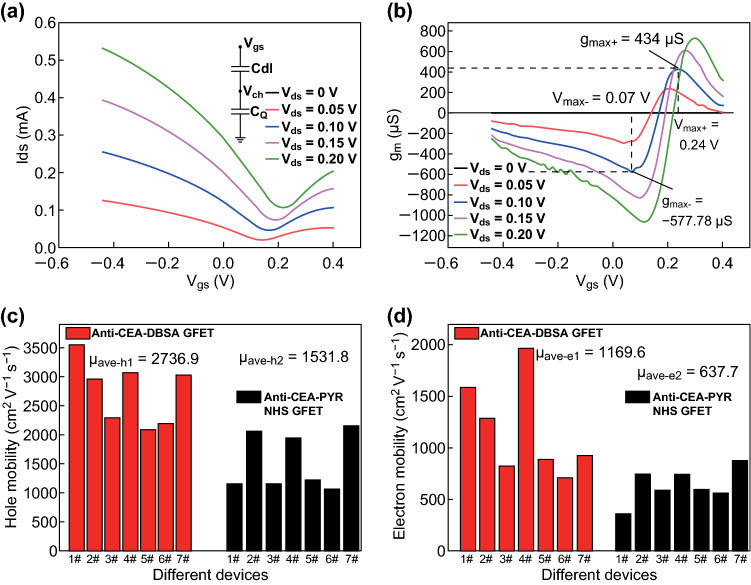
**a** Transfer characteristics of the anti-CEA-dBSA functionalized GFET. The insert in **a** is the capacitive equivalent circuit model of the graphene conducting channel. **b** Anti-CEA-dBSA functionalized GFET at different drain–source voltages was defined as the derivative of Ids with respect to *V*_gs_. **c** Hole mobility of anti-CEA-dBSA functionalized GFET and anti-CEA mAb PYR-NHS functionalized GFET. **d** Electron mobility of anti-CEA-DBSA functionalized GFET and anti-CEA mAb PYR-NHS functionalized GFET

The performances of electrolyte-gated anti-CEA-dBSA functionalized GFETs were evaluated by the fundamental measurements of GFETs. The transfer characteristics were shown in Fig. 3a, which depicted the successful functionalization of nano-dBSA films on graphene and anti-CEAs mAb with the retention of the intrinsic property of graphene. The ambipolar curves indicating the Dirac points (denoted *V*_D_) at *V*_gs_ were between 0.1 and 0.25 V, while *V*_ds_ was below 0.2 V, suggesting that the anti-CEA-dBSA functionalized Gr can be classified as the p-type. A greater difference was observed between two neighboring drain–source currents (*I*_ds_) in the hole regime (the gate–source voltage was denoted *V*_gs_, *V*_gs_ < *V*_D_) than in the electron regime (*V*_gs_ < *V*_D_), indicating that the gate voltage in the hole regime could be a better choice for the detection of target molecules. To maintain the performance of the electrodes and graphene channel, a low *V*_ds_ at 0.1 V was applied to drive the anti-CEA-dBSA functionalized GFET [47].

The transconductance parameter *g*_m_ for a transistor device is widely used to describe FET devices. This parameter represents the amplification capability of GFETs [26, 48], where a higher g_m_ enables a greater conductivity response per unit of biomolecule charge excitation. Therefore, this parameter is positively correlated with the device sensitivity and is valuable for sensing applications. The transconductance *g*_m_ of anti-CEA-dBSA functionalized GFETs under different drain–sources voltages is defined as the derivative of *I*_ds_ with respect to *V*_gs_ in Fig. 3b. While the *V*_ds_ was set at 0.1 V, *g*_m_ = –577.78 μS approached the maximum (denoted *g*_max−_) in the hole regime at a special gate voltage *V*_gs_ = 0.07 V (denoted *V*_max-_). Similarly, at *V*_gs_ = 0.24 V (denoted *V*_max+_), *g*_m_ = 434 μS approached another maximum (denoted *g*_max+_) in the electron regime. The average transconductance value of several anti-CEA-dBSA modified GFETs in Table S1 was higher than that of the anti-CEA mAb PYR-NHS modified GFETs in Table S2 and many other reported electrolyte-gated GFET devices [26, 49, 50], which revealed the high sensitivity of this device for biomolecule detection.

For detailed investigation of the transconductance enhancement mechanism, the hole and electron mobility parameters of the anti-CEA-dBSA functionalized GFETs were calculated according to the transconductance *g*_m_ using Eq. (1) [51]:

1 $$\mu = g_{\text{m}} L/WC_{\text{tot}} V_{\text{ds}}$$

where *L* is the channel length, *W* is the channel width, *C*_tot_ is the gating capacitance per unit channel area (F cm^−2^), *V*_ds_ is the source–drain voltage (*V*), and *g*_m_ is the differential transconductance. For the interfacial capacitance of the graphene–water interface, the quantum capacitance *C*_Q_ of graphene and the double-layer capacitance *C*_dl_ of the electrolyte are in series connection to construct the gate capacitance. Subsequently, according to the capacitive equivalent circuit model of the graphene conducting channel shown in the insert of Fig. 3a, the total gating capacitance per unit area is calculated as: *C*_tot_ = *C*_Q_*C*_dl_/(*C*_Q_ + *C*_dl_). The double-layer capacitance *C*_dl_ acts as a parallel-plate capacitor, which could be calculated using equation: ${C_{\text{dl}}} = {\epsilon_0}{\epsilon_r}/{d_{\text{dl}}}$, where ${\epsilon_0}$ is the permittivity of free space, ${\epsilon_r}$ is the dielectric constant of the electrolyte (~ 78), and *d*_dl_ is the Debye length on the bio-interface. According to the buffer ionic strength of the electrolyte, the Debye length is estimated to be approximately 23 nm, and the corresponding double-layer capacitance *C*_dl_ is approximately 2.97 μF cm^−2^. While the graphene channel potential is *V*_ch_, the quantum capacitance *C*_Q_ of Gr is defined as [26, 52]:

2 $$C_{Q} = \frac{{8\pi q^{2} k_{B} T}}{{\left( {hv_{\text{F}} } \right)^{2} }}\ln \left[ {2\left( {1 + \cosh \frac{{qV_{\text{ch}} }}{{k_{\text{B}} T}}} \right)} \right],$$

where *q* = − 1.602 × 10^−19^ *C* is the electron charge, *K*_B_ = 1.381 × 10^−23^ J K^−1^ is the Boltzmann constant, *h* = 6.626 × 10^−34^ JS is the Planck constant, *v*_F_ = 1.1 × 10^6^ m s^−1^ is the Fermi velocity of Dirac fermions, and *T* = 300 K at room temperature. The potential distribution in the electrolyte-gated anti-CEA-dBSA modified GFET device is described as Eq. (3) [53]:

3 $$C_{Q} /C_{\text{dl}} = \left( {V_{\text{gs}} - V_{\text{ch}} } \right)/V_{\text{ch}} ,$$

where *V*_gs_ is the gate–source voltage. Thus, the *C*_Q_ value at an arbitrarily given *V*_gs_ can be analytically determined by substituting Eq. (3) into Eq. (2) and solving for *C*_Q_.

Using this model for the interfacial capacitance, the field-effect mobility of charge carriers in the device can be obtained. The mobility values extracted at the transconductance maximum points (*g*_max−_ for holes, *g*_max+_ for electrons) were used as the mobility parameters of anti-CEA mAb modified GFET devices. Average values of hole mobility *μ*_ave-h1_ and electron mobility *μ*_ave-e1_ for seven anti-CEA-dBSA GFET devices were estimated to be approximately 2763.9 and 1169.6 cm^2^ V^−1^ s^−1^, respectively. As shown in Fig. 3c, d, the average mobility parameters of several anti-CEA mAb functionalized GFET devices based on a noncovalent functionalized linker (PYR-NHS) were lower than those of anti-CEA-dBSA GFET devices. These results indicated that GFET biosensors based on this multifunctional and self-protecting dBSA film could improve the performance of GFET biosensors.

### Performance of Anti-CEA-dBSA Functionalized GFET

**Fig. 4:**
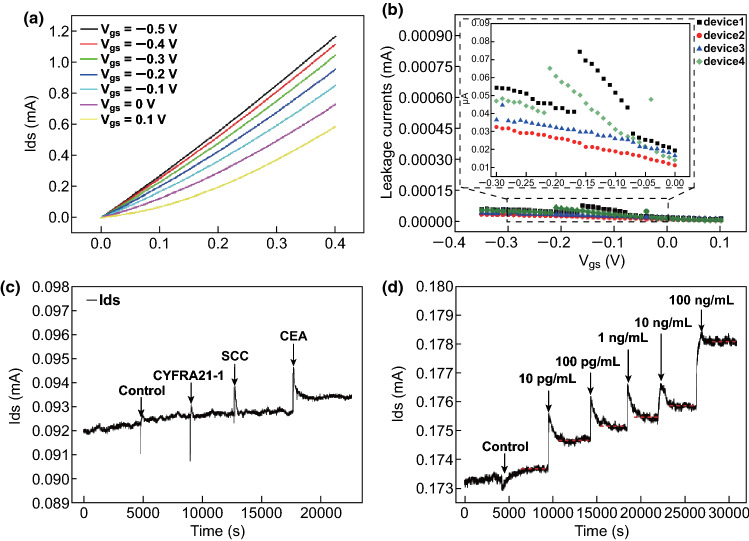
**a** Output characteristics of the anti-CEA-dBSA functionalized GFET. **b** Leakage current of the anti-CEA-dBSA functionalized GFET. **c** Drain–source current curve responses to control group, CYFRA21-1, SCC, and CEA. **d** Monitoring of drain–source current with increasing CEA concentrations (10 pg mL^−1^, 100 pg mL^−1^, 1 ng mL^−1^, 10 ng mL^−1^, and 100 ng mL^−1^)

The output characteristic curves were obtained by recording the *I*_ds_ versus *V*_ds_ under different *V*_gs_, as shown in Fig. 4a. The dependence of the *I*_ds_ with *V*_ds_ variation (− 0.5–0.1 V) verified the good electrical contact between the graphene and gold electrode. The leakage currents were recorded under different top gate voltages (Fig. 4b). Compared with the values of the net change in drain currents, the absolute values of leakage currents were always below 80 nA, which could be considered negligible. To preclude false signals, especially those arising from nonspecific binding, several control groups were used to assess the utility of the anti-CEA-dBSA functionalized GFET. The responses of the drain–source current (*I*_ds_) after adding the same value (10 ng mL^−1^) of control, cytokeratin-19-fragment (CYFRA21-1), SCC, and CEA are shown in Fig. 4c. The general serum diluent was used to dilute the biomarkers, which served as the control group at the same time. As shown in the specific detection curves of the anti-CEA mAb functionalized GFET in Fig. 4c, when the control group, CYFRA21-1, and SCC were added to the buffer solution of the anti-CEA mAb functionalized GFET, no obvious increase was shown in *I*_ds_. Upon the addition of the CEA protein, a large increase in drain current caused by the binding of CEA was observed. To accelerate the reaction between the anti-CEA mAb and CEA protein, the solution was stirred for several seconds after the addition of each protein. The isoelectric point of CEA was approximately 4.4–4.7 [54], indicating that these target molecules were negatively charged in the nearly neutral pH buffer solution. These results demonstrated that the negatively charged CEA protein was avidly bound by the anti-CEA-dBSA functionalized GFET, resulting in an increase in the drain–source current upon addition of CEA. Interestingly, the addition of control proteins SCC and CYFRA21-1 did not induce a similar increase in drain–source current, which indicated that the nonspecific binding of dBSA functionalized graphene with nontarget proteins could be negligible. Taken together, the findings indicated that anti-CEA mAb functionalized GFETs exhibited good specificity for the detection of CEA.

The drain–source current of the anti-CEA-dBSA functionalized GFET was monitored at various CEA protein concentrations to evaluate its sensing characteristics. The target CEA proteins at concentrations of 10 pg mL^−1^, 100 pg mL^−1^, 1 ng mL^−1^, 10 ng mL^−1^, and 100 ng mL^−1^ were introduced into the channel of the anti-CEA-dBSA functionalized GFET as the time-dependent response of the drain current was recorded (Fig. 4d). The mechanism of detection for anti-CEA-dBSA functionalized GFETs involved the adsorption of negative CEA proteins on the surface of the graphene. These proteins acted as electron donors, resulting in conductance changes. For this reason, the drain–source current increased gradually after injection of the target CEA at each concentration (Fig. 4d). According to the response of the control group, the limit of detection was less than 56 fM.

### Target Detection in Diluted Serum Samples

**Fig. 5:**
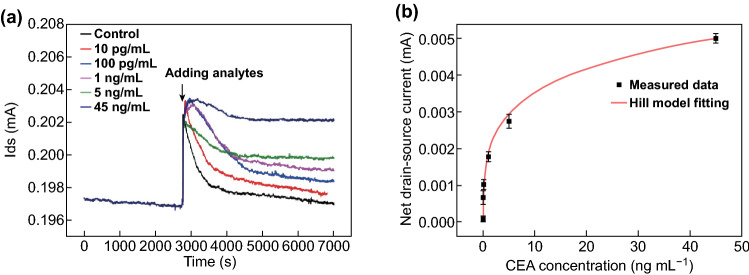
**a** Drain–source current of the anti-CEA-dBSA functionalized GFET in the presence of CEA concentrations of 10 pg mL^−1^, 100 pg mL^−1^, 1 ng mL^−1^, 5 ng mL^−1^, and 45 ng mL^−1^. **b** Net drain–source current at different CEA concentrations fitted with a curve (red line) of the Hill adsorption model. (Color figure online)

Analysis of clinically relevant samples, such as blood serum, could be very important in the clinical diagnosis of cancer. To verify target detection in serum samples using the anti-CEA-dBSA functionalized GFET, the target CEA proteins in diluted blood serum at concentrations of 10 pg mL^−1^, 100 pg mL^−1^, 1 ng mL^−1^, 5 ng mL^−1^, and 45 ng mL^−1^ were added to the reactive chamber, and the drain–source current was recorded at the same time. As shown in Fig. 5a, the drain–source currents increased with the target molecule concentrations. The gradually increasing drain–source current response with increasing CEA concentration in blood serum was consistent with the results in Fig. 4d. The general serum diluent was used to dilute the CEA-containing serum sample, which also worked as a control group.

The average net drain–source currents of the anti-CEA-dBSA functionalized GFET caused by the control, 10 pg mL^−1^, 100 pg mL^−1^, 1 ng mL^−1^, 5 ng mL^−1^, and 45 ng mL^−1^ groups were 0.0747, 0.661, 1.01, 1.77, 2.73, and 4.99 μA, respectively. The dissociation constant (*K*_d_) for the interaction between the anti-CEA mAb and CEA could be estimated by measuring the drain current (*I*_ds_) of the anti-CEA-dBSA functionalized GFET at different CEA concentrations. The quantity of net drain–source current (∆*I*_ds_) was calculated as a function of CEA protein concentrations, as shown in Fig. 5b. The plot of the data yielded a nonlinear curve, indicating that the relationship between ∆*I*_ds_ and the binding CEA could fit the Hill adsorption model [55, 56] calculated as Eq. (4):

4 $$\Delta I =\Delta I_{\hbox{max} } C_{\text{cea}}^{n} /\left( {K_{d}^{n} + C_{\text{cea}}^{n} } \right),$$

where *K*_d_ is the dissociation constant of the interaction between CEA and anti-CEA mAb, Δ*I*_max_ is the saturated net drain–source current, *C*_cea_ is the protein concentration, and n is the Hill cooperativity coefficient of the binding interaction.

According to the fitted red curve shown in Fig. 5b, Δ*I*_max_, *K*_d_, and *n* were estimated to be 12.1 µA, 122.8 ng mL^−1^, and 0.35, respectively. The calculated value of n was less than 1, which indicated the negative cooperativity in binding interaction between CEA and anti-CEA mAb. The molecular weight of CEA of approximately 180 kD [57] resulted in a dissociation constant of 6.82 × 10^−10^ M. The dissociation constant between CEA and anti-CEA mAb had been investigated previously [58, 59], and it was determined to vary from 4 × 10^−12^ to 1 × 10^−7^ M. Therefore, the value of the resulting dissociation constant evaluated in this study using anti-CEA-dBSA functionalized GFETs was in accordance with previously reported results, indicating a high affinity between CEA and anti-CEA mAb. From Eq. (4) and the definition of the dissociation constant (*K*_d_) [60], while the ligand concentration was equal to the dissociation constant (*K*_d_), the percentage of bound receptors at equilibrium was 50%. According to the calculated value (122.8 ng mL^−1^) of K_d_, the available receptors on the dBSA functionalized GFETs bio-interface were sufficient for the detection of CEA molecules under different concentrations in this study. According to the fitting results, the limit of detection was estimated to be approximately 337.58 fg mL^−1^, which was lower than for other graphene FET biosensors [41, 61, 62]. Well-defined drain–source current changes were observed for low CEA concentrations (337.58 fg mL^−1^) in diluted serum, which were much smaller than the cutoff value (5 ng mL^−1^) used in clinical diagnosis. In addition, compared with other nanomaterial-based CEA immunosensors in Table S3, the sensitivity of multifunctional dBSA functionalized GFETs showed obvious superiority. These results clearly demonstrated the promising potential of anti-CEA-dBSA functionalized GFETs in clinical applications.

## Conclusions

A simple, convenient, and sensitive graphene–protein bioelectronic interface for GFETs based on a multifunctional nano-dBSA functionalized process was designed to target cancer biomarkers in diluted serum. This multifunctional nano-dBSA film formed on graphene acted as a protective layer and maintained the electronic properties of graphene during the fabrication of GFET devices and also served as a bifunctional cross-linker to bioconjugate anti-CEA mAb to detect CEA. This novel fabrication process made a high-performance GFET biosensor possible, as evidenced by electronic and fluorescent characterization. Good specificity and ultrahigh sensitivity (337.58 fg mL^−1^) toward CEA molecules were achieved by the measurement of drain–source currents of anti-CEA mAb functionalized GFETs. Measured responses with different orders of magnitude in analytes concentration displayed a good fit to a model based on the Hill binding equation, which indicated the negative cooperativity and a strong affinity between CEA and anti-CEA mAb binding interaction. Experimental results verified that the sensor response was derived from specific binding of the receptor to CEA, indicating that this multifunctional nano-dBSA film maintained its biologically active analyte-binding configuration when noncovalently bound to graphene. By functionalizing such different 2D nanomaterials with related receptors by this nano-dBSA process, it should be possible to offer controllable functionalization methods for various bio-interfaces for biosensors, nanomedicine, imaging, cancer therapy, and drug delivery.

## Electronic supplementary material

Below is the link to the electronic supplementary material.

Supplementary material 1 (PDF 333 kb)
