# Supplementary material for: Novel Graphene Biosensor Based on the Functionalization of Multifunctional Nano-bovine Serum Albumin for the Highly Sensitive Detection of Cancer Biomarkers
Source: Nanomicro Lett. 2019 Mar 9;11:20. doi: 10.1007/s40820-019-0250-8 (PMC7770693; doi:10.1007/s40820-019-0250-8)
Supplement: Supplementary file 1 — Supplementary material 1 (PDF 333 kb) [file 40820_2019_250_MOESM1_ESM.pdf]

Supporting Information for

Novel Graphene Biosensor Based on the Functionalization of Multifunctional Nano-Bovine Serum Albumin for the Highly Sensitive Detection of Cancer Biomarkers

Lin Zhou[1, #], Kun Wang[1, #], Hao Sun[1], Simin Zhao[2], Xianfeng Chen[3], Dahong Qian[2], Hongju Mao[1, *], Jianlong Zhao[1, *]

[1]State Key Laboratory of Transducer Technology; Key Laboratory of Terahertz Solid-State Technology, Shanghai Institute of Microsystem and Information Technology, Chinese Academy of Sciences, Shanghai 200050, People's Republic of China

[2]School of Biomedical Engineering, Shanghai Jiao Tong University, Shanghai 200240, China

[3]School of Electronic Engineering, Bangor University, Bangor LL57 1UT, UK.

[#]These authors contributed equally to this work.

*Corresponding authors. E-mail: hjmao@mail.sim.ac.cn (Hongju Mao), jlzhao@mail.sim.ac.cn (Jianlong Zhao)

S1 The Thickness Characterization of Denatured BSA Functionalized Graphene Channel

The thickness of uniform heat denatured BSA (dBSA) film on single layer graphene channel was measured by optical characterization methods (Lecia DCM8). The thickness of denatured BSA functionalized Gr channel was approximately 26.4 nm when measured along the vertical dash line indicated in Fig. S1a.

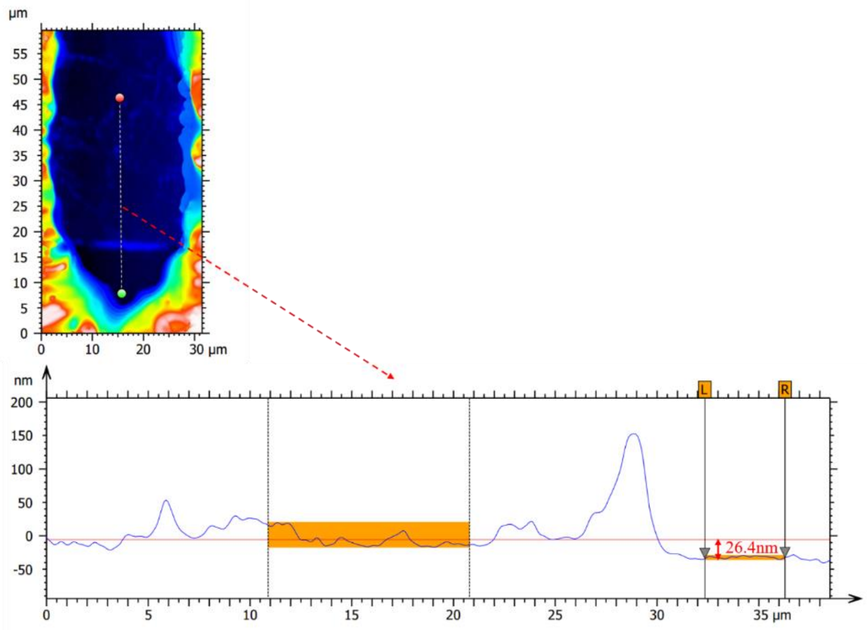

Fig. S1 a Topographic image of the denatured BSA functionalized Gr channel. **b** The thickness of dBSA functionalized Gr channel was approximately 26.4 nm when measured along the vertical dash line indicated in topographic image **a**

S2 Fluorescent Characterization of Anti-CEA-dBSA Functionalized Graphene

Sandwich fluorescent immunoassay was used to verify the successful immobilization of anti-CEA on dBSA here. Secondary anti-CEA labeled with quantum dots (QDs) with the concentration of 100nM mixed with 100 ng mL^{-1} CEA solution. Then this mixed solution incubated with anti-CEA-dBSA functionalized graphene and bare dBSA functionalized graphene for one hour, respectively. Then fluorescent images of anti-CEA-dBSA functionalized graphene and bare dBSA functionalized graphene were shown in Fig. S2a, b, while the time of exposure is 5.5 s.

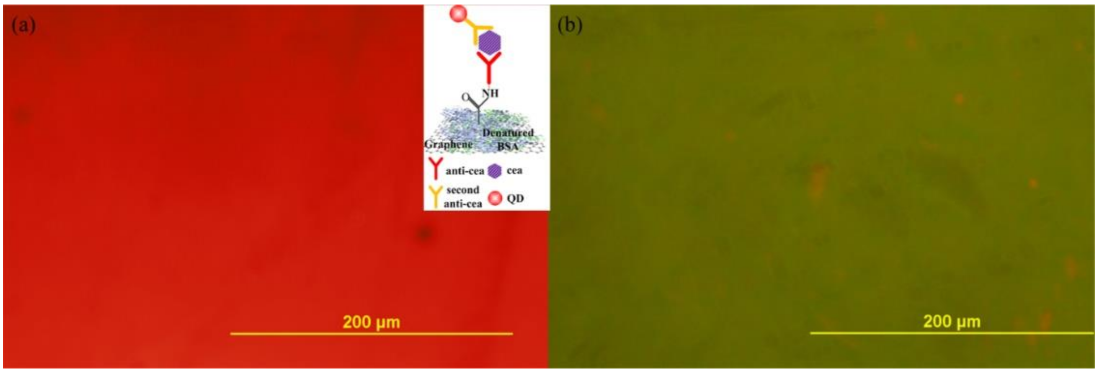

Fig. S2 a Fluorescent image of anti-CEA-dBSA functionalized graphene (The insert is the schematic diagram of sandwich fluorescent immunoassay of dBSA modified graphene). **b** fluorescent image of bare dBSA functionalized graphene

S3 Comparison of the Transconductance, Gating Capacitance and Mobility Parameters of Electrolyte-Gated Anti-CEA-dBSA Functionalized GFET and anti-CEA-PYR NHS Functionalized GFET

The complete experimental data of the transfer characteristics, the corresponding transconductance, the calculated total gating capacitance and mobility were presented in this section. Allof the experiments were conducted in 0.001× PBS buffer and the drain-source voltage (V_{ds}) was set at 0.1 V. The double layer capacitance C_{dl} of the electrolyte was 2.97 μF cm^{-2}.

Table S1 Transconductance, gating capacitance and mobility parameters of electrolyte-gated anti-CEA-dBSA functionalized GFET devices

Device	V_D (V)	Hole regime					Electron regime				
		V_{max-} (V)	g_{max-}	C_Q (uF cm^{-2})	C_{tot} (uF cm^{-2})	μ_h (cm^2 V^{-1}s^{-1})	V_{max+} (V)	g_{max+} (μS)	C_Q (uF cm^{-2})	C_{tot} (uF cm^{-2})	μ_e (cm^2 V^{-1}s^{-1})
1#	0.17	0.07	-577.78	1.1205	0.8136	3550.9	0.24	434	2.5283	1.3657	1588.9
2#	0.17	0.08	-509.05	1.2113	0.8604	2958.2	0.24	352.43	2.5283	1.3657	1290.3
3#	0.17	0.06	-350.65	1.0316	0.76566	2289.9	0.22	217.31	2.3831	1.3222	821.8
4#	0.14	-0.07	-499.93	1.1205	0.8136	3072.4	0.26	553.44	2.6687	1.4057	1968.6
5#	0.19	0.11	-411.66	1.4840	0.9896	2080.0	0.25	245.55	2.5991	1.3861	885.8
6#	0.22	0.07	-354.57	1.1205	0.8136	2179.1	0.29	205.91	2.8714	1.4599	705.2
7#	0.17	0.07	-492.66	1.1205	0.8136	3027.8	0.25	256.89	2.5991	1.3861	926.7

Table S2 Transconductance, gating capacitance and mobility parameters of electrolyte-gated anti-CEA-PYR NHS modified GFET devices

Device	V_D (V)	Hole regime					Electron regime				
		V_{max-} (V)	g_{max-} (μS)	C_Q (uF cm^{-2})	C_{tot} (uF cm^{-2})	μ_h (cm^2 V^{-1}s^{-1})	V_{max+} (V)	g_{max+} (μS)	C_Q (uF cm^{-2})	C_{tot} (uF cm^{-2})	μ_e (cm^2 V^{-1}s^{-1})
1#	0.38	0.18	-70.281	2.0769	1.2222	1150.1	0.7	33.2488	5.0452	1.8695	355.7
2#	0.52	0.26	-144.922	2.6687	1.4057	2062.0	0.78	71.1832	5.3900	1.9149	743.5
3#	0.36	0.2	-73.8079	2.2328	1.2746	1158.1	0.64	53.532	4.7742	1.8310	584.7
4#	0.26	0.105	-58.7013	1.4390	0.9693	1211.2	0.5	51.179	4.0910	1.7208	594.8
5#	0.27	0.03	-61.0435	0.7990	0.6296	1939.1	0.59	66.676	4.5391	1.7953	742.8
6#	0.33	0.18	-131.129	2.0769	1.2222	2145.8	0.78	84.3242	5.3900	1.9149	880.7
7#	0.29	0.165	-62.305	1.9562	1.1794	1056.5	0.4	45.3665	3.5468	1.6164	561.3

S4 Comparison with the Sensitivity of Other Nanomaterial-Based Immunosensors for CEA Detection

Compared to other nanomaterial-based immunosensors in the below table, the sensitivity of multifunctional dBSA modified GFETs was 337.58 fg mL^{-1}, which showed obvious superiority.

Table S3 The sensitivity of nanomaterial-based immunosensors for CEA detection

Sensing element	Method	Limit of Detection (LOD, ng mL^{-1})	Refs.
Graphene nanocomposites	Electrochemical	0.1	[S1]
Paper-based microfluidic electrochemical immunodevice	Electrochemical	0.01	[S2]
graphene oxide/carboxylated multiwall carbon nanotubes/gold/cerium oxide nanoparticles	Electrochemiluminescence	0.02	[S3]
porphyrin-sensitized titanium dioxide (TiO2) nanostructures	Photoelectrochemical immunoassay	0.006	[S4]
aptamer/graphene oxide	Capillary electrophoresis-chemiluminescence	0.0048	[S5]
gold nanoparticles	Immunochromatography test strip	0.0059	[S6]
magnetic FICTS --PLGA@Fe_3O_4 super-paramagnetic nanosphere	Immunochromatography test strips	0.06	[S7]
magnetic nanoparticle/ bimetallic nanoparticles	Electrochemical immunosensor	4.31	[S8]
silver nanoclusters (AgNCs) pair	Ratiometric fluorescence	0.1	[S9]
AgNCs@Apt@UiO-66	Electrochemical/SPR	0.0049	[S10]
magnetic bead--Au NP/urease nanoprobe	Colorimetric	0.00045	[S11]
CVD grown graphene	Electrochemical	0.23	[S12]
Concanavalin A/ HRP	Electrochemical	3.4	[S13]
Silicon nanowire arrays	FET	0.000001	[S14]
silicon nanoribbon	FET	0.1	[S15]
**dBSA--Graphene**	**FET**	**0.000337**	**This work**

Supplementary References

[S1] X. Chen, X.L. Jia, J. M. Han, J. Ma, Z.F. Ma, Electrochemical immunosensor for simultaneous detection of multiplex cancer biomarkers based on graphene nanocomposites. Biosens. Bioelectron. **50**, 356-361 (2013). https://doi.org/10.1016/j.bios.2013.06.054

[S2] Y.F. Wu, P. Xue, K.M. Hui, Y.J. Rang, A paper-based microfluidic electrochemical immunodevice integrated with amplification-by-polymerization for the ultrasensitive multiplexed detection of cancer biomarkers. Biosens. Bioelectron. **52**, 180-187 (2014). https://doi.org/10.1016/j.bios.2013.08.039

[S3] X. Pang, J. Li, Y. Zhao, D. Wu, Y. Zhang, B. Du, H. Ma, Q. Wei, Label-free electrochemiluminescent immunosensor for detection of carcinoembryonic antigen based on nanocomposites of GO/MWCNTs-COOH/Au@CeO_2. ACS Appl. Mater. Interfaces **7**(34), 19260-19267 (2015). https://doi.org/10.1021/acsami.5b05185

[S4] J. Shu, Z. Qiu, J. Zhuang, M. Xu, D. Tang, In situ generation of electron donor to assist signal amplification on porphyrin-sensitized titanium dioxide nanostructures for ultrasensitive photoelectrochemical immunoassay. ACS Appl. Mater. Interfaces **7**(42), 23812-23818 (2015). https://doi.org/10.1021/acsami.5b08742

[S5] Z.M. Zhou, Z. Feng, J. Zhou, B.Y. Fang, X.X. Qi et al., Capillary electrophoresis-chemiluminescence detection for carcino-embryonic antigen based on aptamer/graphene oxide structure. Biosens. Bioelectron. **64**, 493-498 (2015). https://doi.org/10.1016/j.bios.2014.09.050

[S6] Y. Yao, W. Guo, J. Zhang, Y. Wu, W. Fu et al., Reverse Fluorescence enhancement and colorimetric bimodal signal readout immunochromatography test strip for ultrasensitive large-scale screening and postoperative monitoring. ACS Appl. Mater. Interfaces **8**(35), 22963–22970 (2016). https://doi.org/10.1021/acsami.6b08445

[S7] B. Zhang, W. Ma, F. Li, W. Gao, Q. Zhao et al., Fluorescence quenching-based signal amplification on immunochromatography test strips for dual-mode sensing of two biomarkers of breast cancer. Nanoscale **9**, 18711-18722 (2017). https://doi.org/10.1039/C7NR06781J

[S8] D. Kalyoncu, Y. Tepeli, U. Can Kirgöz, A. Buyraç, Ü. Anik, Electro-nano diagnostic platforms for simultaneous detection of multiple cancer biomarkers. Electroanalysis **29**, 2832-2838 (2017). https://doi.org/10.1002/elan.201700556

[S9] K. Wang, M.Q. He, F.H. Zhai, R. H. He, Y.L. Yu, A label-free and enzyme-free ratiometric fluorescence biosensor for sensitive detection of carcinoembryonic antigen based on target-aptamer complex recycling amplification. Sens. Actuat. B **253**, 893-899 (2017). https://doi.org/10.1016/j.snb.2017.07.047

[S10] C. Guo, F. Su, Y. Song, B. Hu, M. Wang, L. He, D. Peng, Z. Zhang, Aptamer-templated silver nanoclusters embedded in zirconium metal–organic framework for bifunctional electrochemical and SPR aptasensors toward carcinoembryonic antigen. ACS Appl. Mater. Interfaces **9** (47), 41188–41199 (2017). https://doi.org/10.1021/acsami.7b14952

[S11] B. Li, G. Lai, B. Lin, A. Yu, N. Yang, Enzyme-induced biomineralization of cupric subcarbonate for ultrasensitive colorimetric immunosensing of carcinoembryonic antigen. Sens. Actuat. B **262**, 789 (2018). https://doi.org/10.1016/j.snb.2018.02.049

[S12] V.K. Singh, S. Kumar, S.K. Pandey, S. Srivastava, M. Mishra, G. Gupta, B.D. Malhotra, R.S. Tiwari, A. Srivastava, Fabrication of sensitive bioelectrode based on atomically thin CVD grown graphene for cancer biomarker detection. Biosens. Bioelectron. **105**, 173-181 (2018). https://doi.org/10.1016/j.bios.2018.01.014

[S13] Q.L. Wang, H.F. Cui, X. Song, S.F. Fan, L.L. Chen, M.M. Li, Z.Y. Li, A label-free and lectin-based sandwich aptasensor for detection of carcinoembryonic antigen. Sens. Actuat. B **260**, 48-54 (2018). https://doi.org/10.1016/j.snb.2017.12.105

[S14] A. Gao, X. Yang, J. Tong, L. Zhou, Y. Wang, J. Zhao, H. Mao, T. Li, Multiplexed detection of lung cancer biomarkers in patients serum with CMOS-compatible silicon nanowire arrays. Biosens. Bioelectron. **91**, 482-488 (2017). https://doi.org/10.1016/j.bios.2016.12.072

[S15] Z. Bao, J. Sun, X. Zhao, Z. Li, S. Cui, Q. Meng, Y. Zhang, T. Wang, Y. Jiang, Top-down nanofabrication of silicon nanoribbon field effect transistor (Si-NR FET) for carcinoembryonic antigen detection. Int. J. Nanomedicine **12**, 4623-4631 (2017). https://doi.org/10.2147/IJN.S135985
